# Misdiagnosis of a Term Abdominal Ectopic Pregnancy: A Case Report

**DOI:** 10.31729/jnma.9074

**Published:** 2025-06-30

**Authors:** Kumar Bahadur Bista, Rakesh Pariyar, Aashis Poudel, Kalpana Kumari Yadav, Sharmila Ghimire

**Affiliations:** 1 Pokhara Academy of Health Sciences, Pokhara, Nepal; 2 Patan Academy of Health Sciences, Lalitpur, Nepal; 3 Institute of Medicine, Tribhuvan University, Kathmandu, Nepal

**Keywords:** *case report*, *ectopic pregnancy*, *misdiagnosis*, *term birth*

## Abstract

Abdominal ectopic pregnancy is a rare and life-threatening condition, often misdiagnosed due to its nonspecific clinical presentation and imaging challenges. We present a case of a 20-year-old primigravida at 39+6 weeks of gestation that was initially misdiagnosed as complete placenta previa with transverse lie and was only identified as abdominal ectopic pregnancy intraoperatively. An emergency cesarean section was planned for suspected placenta previa, during which an intact amniotic sac was found in the peritoneal cavity, with placental attachment to the greater omentum, left ovary, and fallopian tube for which cesarean section was converted to the laparotomy. The baby was live at birth but succumbed to respiratory distress on the third day of life. A multidisciplinary team helped in optimizing maternal outcomes. Early recognition, appropriate imaging, and surgical expertise are essential to reduce morbidity and mortality.

## INTRODUCTION

Abdominal ectopic pregnancy (AEP) has an estimated incidence of 1 in 10,000 to 25,000 live births.^1^ Diagnosing AEP requires a high degree of clinical suspicion since the clinical findings are often nonspecific.^2^ Accurate and timely diagnosis of AEP is crucial to reduce the risks of maternal hemorrhage and perinatal mortality.^1^

We report a case of a term abdominal pregnancy, which was initially misdiagnosed as placenta previa. This case underscores the importance of clinical vigilance and accurate diagnosis to improve outcomes in AEP. We report this case as per SCARE guidelines.^3^

## CASE REPORT

A 20-year-old primigravida at 39 weeks and 6 days of gestation, with a history of regular antenatal visits at a provincial hospital, presented to a tertiary care center for delivery. She was initially misdiagnosed with complete placenta previa with transverse lie by USG. Her last menstrual period was recorded as October 11, 2021, with a history of regular menstrual cycles. During her antenatal care, she underwent five check-ups and was consistently diagnosed with placenta previa and transverse lie. She had no episodes of abdominal pain or vaginal bleeding.

At the presentation, her general condition was fair. Vital signs were stable. She had no pallor and no icteric sclera. Cardiopulmonary examination findings were within normal physiological limits. Abdominal examination revealed a uterus of term size, fetus in transverse lie, no tenderness, and no uterine contractions. The fetal heart rate was 136 bpm. Ultrasonography showed a single live fetus in transverse lie, with an estimated gestational age of 36+6 weeks, a fetal weight of 2900 grams, adequate amniotic fluid, and complete placenta previa.

Per speculum examination showed a blood-stained cervix. After per speculum exam was done, there was no evidence of placenta previa so, it was planned to do double setup examination. Per vaginal examination revealed a cervix that was 1 cm dilated, soft, uneffaced, and posterior, with the presenting part high and a show present.

The patient was admitted and her complete blood count, urine examination, liver and renal function tests, and blood grouping with crossmatch were sent. Hemoglobin was 11.1 g/dl, and the blood group was O positive. Other parameters were within normal limits. In light of her clinical presentation, an emergency cesarean section was planned due to the transverse lie in labor. The patient was counseled, consent was obtained, and prophylactic antibiotics were administered. The procedure was initiated under spinal anesthesia via Pfannenstiel incision.

**Figure 1:**
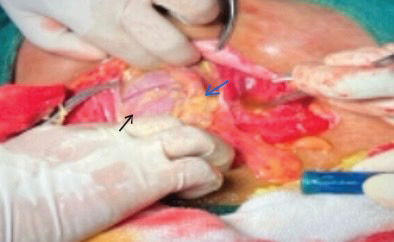
Intraoperative picture of intact amniotic membrane (black arrow) attached to omentum (blue arrow)

Intraoperatively, an intact amniotic sac was identified within the abdominal cavity. The placenta was found adhered to the greater omentum, left ovary, and left fallopian tube. The uterus was bulky and located posterior to the amniotic sac; the right adnexa were normal. The procedure was converted to general anesthesia. The amniotic sac was opened, revealing meconium-stained fluid, and the baby was delivered via breech extraction. We delivered a live female neonate weighing 3000 grams with APGAR scores of 4 and 6 at one and five minutes, respectively. The placenta was separated from the omentum and sent for histopathology, which later confirmed the presence of normal chorionic villi, fibrin, and decidual stroma (Figure 1).

Estimated intraoperative blood loss was approximately 3500 ml. The patient received seven pints of whole blood during and after the surgery and was transferred to the ICU. The neonate was shifted to the NICU due to poor respiratory effort and excessive greenish secretions. Despite intubation and oxygen therapy, the baby succumbed on the third day due to respiratory failure. The mother had an uneventful course and was discharged on the seventh postoperative day.

## DISCUSSION

This case highlights a 20-year-old primigravida presenting with placenta previa and transverse lie in labor, which upon surgical exploration, was found to be an abdominal ectopic pregnancy. Ectopic pregnancies are defined as implantation of a fertilized ovum outside the endometrial cavity. Approximately 95% occur in the fallopian tube, while the remaining 5% are located in the ovary, cervix, cesarean scar, or abdominal cavity.^1^ Abdominal pregnancies represent only 1% of ectopic pregnancies.^4^ Primary abdominal pregnancy involves direct peritoneal implantation, while secondary results from tubal rupture or abortion with reimplantation.^1^

The diagnosis of AEP is often difficult due to its rarity and complex presentation. Ultrasonographic features may include a fetus outside the uterus, absence of uterine wall between the fetus and bladder, abnormal fetal positions, and close proximity of the fetus to the maternal abdominal wall.^5^ However, the diagnostic error rate using ultrasound is notably high, ranging from 50% to 90%.^6^

In this case, repeated ultrasonography failed to identify the abdominal pregnancy, leading to misdiagnosis as placenta previa. This underscores a critical gap in clinical training and underscores the need for ongoing professional development, particularly in the use of obstetric ultrasound. A study by Shakeel et al. found TAS had a sensitivity of 88.5% and specificity of 96.5% on the first scan.^7^ Advanced imaging tools such as MRI can further improve diagnostic accuracy.^1^

Clinical signs such as fetal malpresentation, abnormal cervix location, and labor failure should raise suspicion of AEP.^8^ In pregnancies diagnosed beyond 20 weeks, conservative management may be considered, involving close surveillance and planned delivery.^9^ However, laparotomy remains the definitive treatment due to the risk of hemorrhage associated with placental separation. Intraoperative management of the placenta is a critical determinant of maternal outcome. Many obstetric practices recommend partial removal or leaving the placenta in situ to reduce hemorrhage risk.^10^

In our case, the placenta was attached to the greater omentum and left adnexa. Surgical teams performed adhesiolysis, with controlled hemostasis enabling complete placental removal. The mother tolerated the procedure well despite significant blood loss.

Maternal mortality in AEP ranges between 2% and 18%, while perinatal mortality rates can be as high as 75-95%.^1^ In our case, neonatal demise occurred due to persistent respiratory distress and hypoxia despite intensive care.

Abdominal ectopic pregnancy is associated with high morbidity and mortality, necessitating a multidisciplinary approach for optimal management. Effective treatment requires careful preoperative planning, involving obstetricians, surgeons, anesthesiologists, and critical care specialists to mitigate intraoperative and postoperative risks. Surgical intervention must be meticulously executed, considering factors such as vascular involvement, adhesion severity, and potential complications, to ensure favorable maternal outcomes.
